# Dying in the emergency service: nurses’ attitudes before and after the first critical period of COVID-19

**DOI:** 10.1097/j.pbj.0000000000000149

**Published:** 2022-02-08

**Authors:** Maria Filomena Passos Teixeira Cardoso, Maria Manuela Ferreira Pereira da Silva Martins, João Miguel Almeida Ventura-Silva, Paulo Emílio Mota, Paula Cristina Rodrigues Costa, Olga Maria Pimenta Lopes Ribeiro

**Affiliations:** a Instituto de Ciências Biomédicas Abel Salazar, Universidade do Porto; b Centro Hospitalar Universitário São João; c Universidade Fernando Pessoa; d Escola Superior de Enfermagem do Porto; e Centro de Investigação em Tecnologias e Serviços de Saude – CINTESIS, Porto, Portugal.

**Keywords:** attitude toward death, coronavirus infections, nurses, nursing, pandemic

## Abstract

**Background:**

Death is an increasingly frequent event in hospitals, and nurses are the health professionals who live with this reality the most. The pandemic caused by COVID-19 made this event more present, showing that nurses’ attitudes toward death may influence the care provided to people at the end of life. The objective of this study was to analyze the attitudes of nurses in the emergency service toward death, before and after the first critical period of the pandemic by COVID-19 in Portugal.

**Methods:**

A quantitative, comparative, and cross-sectional study was conducted in a hospital in Northern Portugal at 2 different moments: the first in February 2018 and the second in May 2020, after the first critical period of the pandemic by COVID-19. In both moments, data were collected using a self-completion questionnaire, which included the Death Attitude Profile Assessment Scale.

**Results:**

The attitudes fear, avoidance, closeness, and escape did not show significant differences. In neutral/neutral acceptance, differences were found between the first and second moments of data collection (*P* *=* .01), with a lower mean after the critical period of the pandemic.

**Conclusions:**

The results obtained in 2018 and 2020 showed slight changes in attitudes toward death. The need to invest in the training and preparation of nurses who deal directly with death and the dying process was evident. Nurse managers should promote spaces for reflection and team training on death, aiming to reduce the professionals’ suffering and anxiety.

## Introduction

Death and the dying process are an integral part of the natural cycle of life. For today's society, it is a moment marked by grief, helplessness, frustration, and by nonacceptance of the loss and difficulties in facing it, with associated feelings such as fear, loneliness, grief, anger, and sadness.^1,2^ It is characterized as the end of a cycle, from the moment one is born until one dies, triggering reactions for which science seeks an answer, trying to compensate the pain of loss and overcome the irreversibility of death.^3^

For nurses who provide direct care to the person, death is part of their daily lives, generating unequal reactions and involving different attitudes and feelings.^4^

In the emergency service (ES), the professional practice of nurses is directed toward the identification of cases of severe instability, early detection of complications or resuscitation, and there may also be situations of end-of-life care and, consequently, the death of the person.^5^

The year 2020 was marked by the SARS-CoV-2 infection and the need to create and reinvent new strategies, such as the reorganization of materials/structures and human resource management, to better respond to the need for care that these patients require.^6–8^ This pandemic contributed to an increase in mortality in Portugal, being responsible for 6972 deaths in 2020.^9^ In fact, nurses were also confronted with this reality in the ES, with the presence of different dynamics in the monitoring of death, which until then was shared by the presence of family members. The suspension of visits and, consequently, the absence of good-byes at this moment, were a reality experienced by many nurses, who inevitably became emotionally involved with the anguish of the patients and their relatives.^10^ It is important to note that in patients with COVID-19, the rapid and unexpected onset of illness,^11^ as well as the fact that it affects patients at a young age, makes it even more difficult to experience death.

It is necessary to search for the aspects that may determine the actions of nurses in the ES, starting by knowing their attitudes toward death. These attitudes can be classified as positive (acceptance as Approach, neutral/neutral acceptance, and acceptance as escape) or negative (fear and avoidance).^12^

Given this reality and the characteristics/events inherent to the pandemic by COVID-19, it is important to question whether there are differences in the attitudes of nurses working in the ES toward the death of patients before and after the first critical period of the pandemic.

Considering our concern, as part of a larger research project initiated in 2017, the aim of this study is to analyze the attitudes of ER nurses toward death, before and after the first critical period of the pandemic by COVID-19.

## Method

This is a quantitative, comparative, and cross-sectional study carried out in a Hospital Center in Northern Portugal. Data were collected in 2 different moments: the first in February 2018 and the second in May 2020, after the first critical period of the pandemic by COVID-19. An unpaired convenience sample was used, composed of all nurses working in the ES at the time of data collection. In a universe of 113 nurses, there was a sample of 81 participants at the first moment and 85 at the second moment.

**Figure 1:**
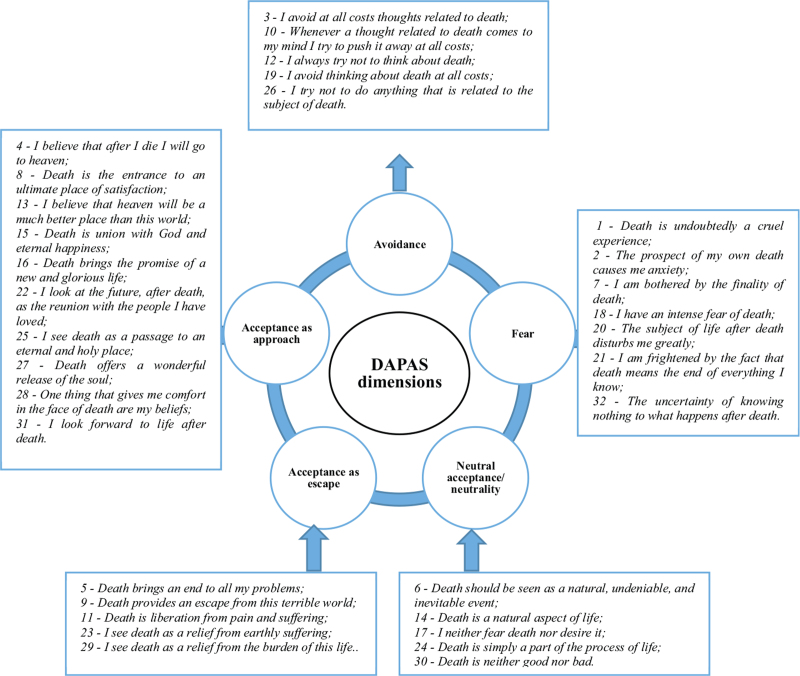
Dimensions and items of DAPAS. DAPAS = Death Attitude Profile Assessment Scale.

As an instrument for data collection, a self-completed questionnaire was used, consisting of 2 parts: the first with the nurses’ sociodemographic and professional characterization, and the second with the Death Attitude Profile Assessment Scale (DAPAS), which encompasses 5 dimensions and 32 items (Fig. 1).

In DAPAS, the “fear” dimension refers to thoughts, feelings, and fear about death. The “avoidance” dimension relates to avoiding or thinking about death. In the “acceptance” dimension, neutral/neutral acceptance understands death as an integral part of life; acceptance as Approach is directed toward the religious dimension and beliefs; acceptance as escape is presented as an alternative to the ending of pain and suffering.^12^

The response to each item is scored on a Likert-type scale ranging from 1 (strongly disagree), 2 (disagree), 3 (moderately disagree), 4 (neither agree nor disagree), 5 (moderately agree), 6 (agree), and 7 (strongly agree). The total DAPAS score can range from 32 (all responses scored at 1) to 224 (all responses scored at 7). For the study sample, the DAPAS obtained a Cronbach alpha of 0.880.

**Figure 2:**
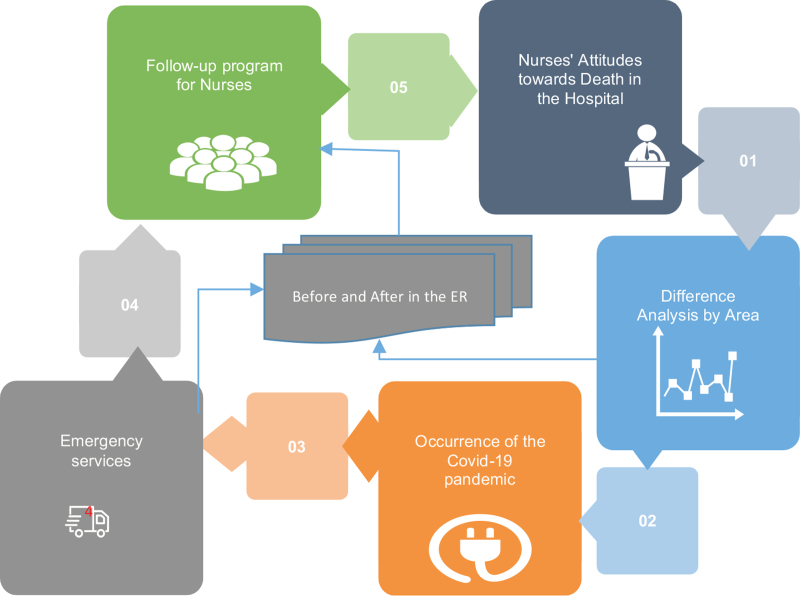
Study design.

The research is part of a project developed in several services, to create a follow-up program for nurses, considering the attitudes they develop toward death (Fig. 2).

This study had the favorable opinion of the ethics committee of the Hospital Center where it took place, with the number 102/2017 and extension addendum of the work approved on May 26, 2020.

Data collection was performed by one of the researchers, delivering in the ER the questionnaires corresponding to the number of nurses. Subsequently, they were collected on site, according to the professionals’ availability. Participants were informed about the study objectives, as well as about their voluntary participation, and had the possibility of giving up at any time, without incurring any prejudice. The nurses signed the informed consent form, being guaranteed anonymity and data confidentiality in the various moments of dissemination of results.

Data analysis was performed through descriptive and inferential statistics, using the Statistical Package for the Social Sciences, version 22.0. To ascertain the nurses’ agreement or disagreement with the items of the DAPAS, we elected as strategy that disagreement resulted from the sum of the items “strongly disagree” (1), “disagree” (2) and “moderately disagree” (3), and agreement from the sum of the items “moderately agree” (5), “agree” (6), and “completely agree” (7). Student *t* test was used to check for statistically significant differences (*P* *<* .05) between the scale dimensions before and after the first critical period of the pandemic by COVID-19.

## Results

Regarding sociodemographic and professional characterization, the 81 nurses who participated in the first moment were predominantly women (66.67%; n = 54), aged between 26 and 35 years (49.38%; n = 40) and mostly married/living in a consensual union (48.15%; n = 39). As regards the professional category, the highest number of answers were given by nurses (82.72%; n = 67) and specialist nurses (17.28%; n = 14). In the area of specialization, medical-surgical nursing stood out (78.57%; n = 11), followed by rehabilitation nursing (21.43%; n = 3).

In the second moment of data collection, the 85 nurses were also mostly female (69.41%; n = 59), aged between 36 and 45 years (49.41%; n = 42) and mostly married/living together (57.65%; n = 49). In terms of professional category, most participants were nurses (83.53%; n = 71) and specialist nurses (12.94%; n = 11), from the specialty areas of medical-surgical nursing (63.64%; n = 7) and community health nursing (18.18%; n = 2). Most participants were on duty at the second moment of data collection (90.59%; n = 77), and, in the case of absence from work, the reason was SARS-CoV-2 infection (50.00%; n = 4), followed by maternity leave (25.00%; n = 2).

**Graph 1:**
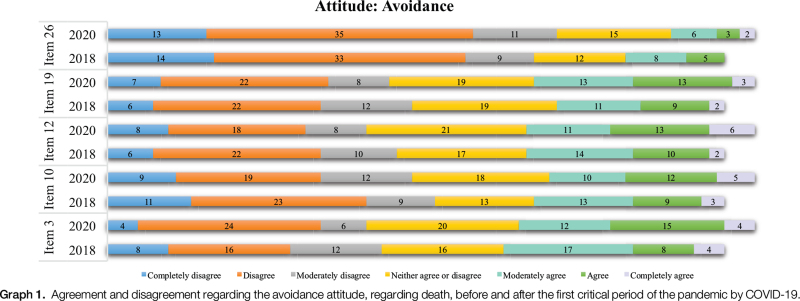
Agreement and disagreement regarding the avoidance attitude, regarding death, before and after the first critical period of the pandemic by COVID-19.

In the analysis of participants’ agreement and disagreement regarding the avoidance attitude (Graph 1) in the data collection periods, we found that item 3, “I avoid death-related thoughts at all costs,” and item 12, “I always try not to think about death,” showed the highest levels of agreement. In both periods, the highest levels of disagreement were found in items 26 and 10, respectively, “I try not to do anything related to the subject of death” and “Whenever a thought related to death comes to my mind, I try to push it away at all costs.”

**Graph 2:**
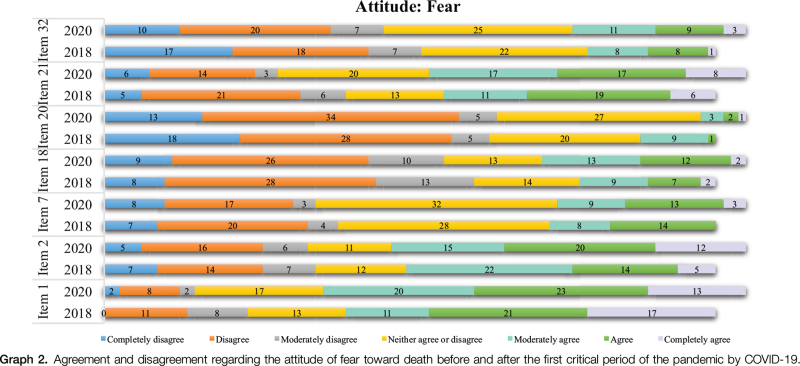
Agreement and disagreement regarding the attitude of fear toward death before and after the first critical period of the pandemic by COVID-19.

In the attitude of fear (Graph 2), the nurses, in 2018 and 2020, showed a high level of agreement in items 1 and 2, respectively, “Death is undoubtedly a cruel experience” and “The prospect of my own death causes me anxiety.” Disagreement, in the same periods, was notable in the items “The issue of life after death disturbs me a lot” (item 20) and “I have an intense fear of death” (item 18).

**Graph 3:**
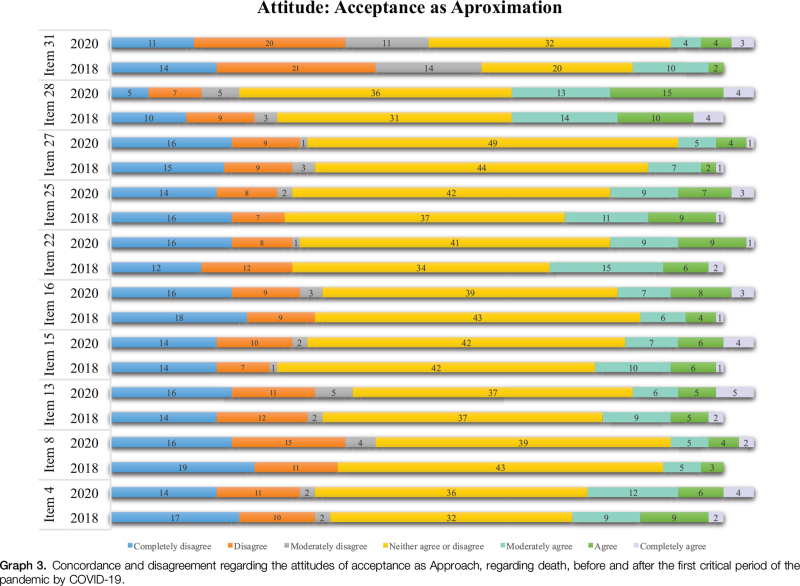
Concordance and disagreement regarding the attitudes of acceptance as Approach, regarding death, before and after the first critical period of the pandemic by COVID-19.

In the various dimensions of the Acceptance attitude, in Acceptance as Approach (Graph 3), it was found that in 2018 the nurses’ highest agreement was reflected in item 28 “One thing that gives me comfort in the face of death are my beliefs” and in item 22 “I look to the future, after death, as the reunion with the people I loved.” In the year 2020, there was a slight change in perspective. Although the highest value of agreement of the participants was also found in item 28, the second highest value was found in item 4 “I believe that after I die I will go to heaven.” Also in this attitude, the nurses’ highest levels of disagreement, in both moments, were found in items 31 and 8, respectively, “I look forward to life after death” and “Death is the entrance into an ultimate place of satisfaction.”

**Graph 4:**
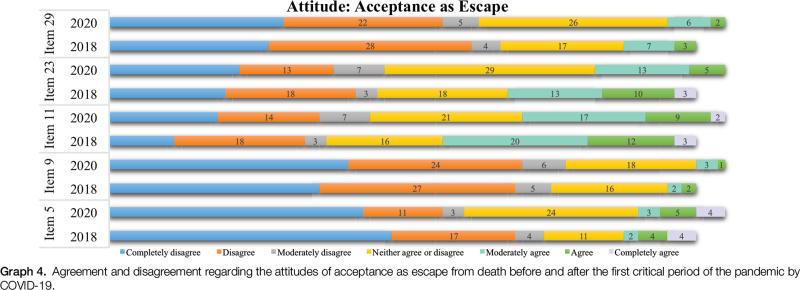
Agreement and disagreement regarding the attitudes of acceptance as escape from death before and after the first critical period of the pandemic by COVID-19.

In Acceptance as an Escape, in both periods, the highest agreement among participants was in the statements “Death is liberation from pain and suffering” (item 11) and “I see death as a relief from earthly suffering” (item 23). In 2018, the highest disagreement was found in item 9, “Death provides an escape from this terrible world,” followed by item 5, “Death brings an end to all my problems,” whereas in 2020 disagreement was highest in items 9, “Death provides an escape from this terrible world,” and 29, “I see death as relief from the burden of this life” (Graph 4).

**Graph 5:**
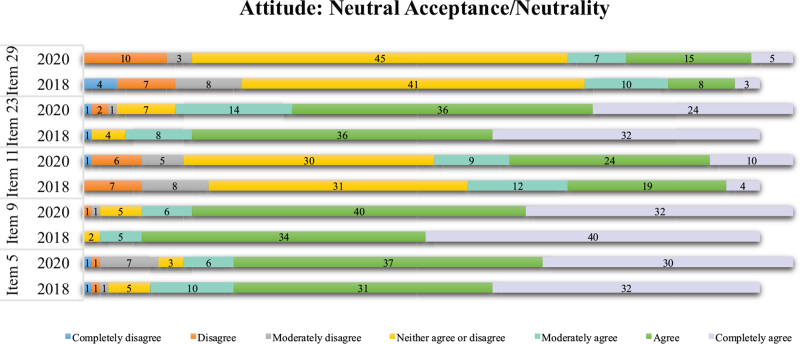
Agreement and disagreement regarding neutral/neutral acceptance attitudes toward death before and after the first critical period of the pandemic by COVID-19.

In the Neutral Acceptance/Neutrality attitude (Graph 5), there was a high level of agreement in both data collection periods regarding item 24, “Death is simply a part of the life process,” and item 14, “Death is a natural aspect of life.” The highest disagreement, in both periods, focused on items 30 and 17, respectively, “Death is neither good nor bad” and “I neither fear death nor desire it.”

Using the Student t test, it is possible to observe that, for a significance level of 5% (*P* < .05), only the Neutral Acceptance/ Neutrality attitude shows statistically significant differences (*P* = .01), with a higher mean in 2018. In the dimensions fear, avoidance, acceptance as Approach and as escape, there were no statistically significant changes. It should be added that the mean obtained in these dimensions was always higher in the second moment of data collection, except for the attitude Acceptance as an Escape, which remained slightly overlapping in 2018 and 2020 (Table 1).

**Table 1:** Results of the Death Attitude Profile Assessment Scale dimensions, at the 2 moments of data collection.

DAPAS/dimensions	Time of data collection	n	Mean	Standard deviation	Mean comparison test
Total Scale Value	2018	81	119.00	23.63	0.78^∗^
	2020	85	122.61	24.33	
Fear	2018	81	25.81	7.95	0.47^∗^
	2020	85	27.21	8.45	
Avoidance	2018	81	17.02	6.45	0.56^∗^
	2020	85	17.88	6.97	
Acceptance as Approach	2018	81	34.62	12.449	0.87^∗^
	2020	85	35.73	12.87	
Acceptance as Escape	2018	81	14.52	6.021	0.66^∗^
	2020	85	14.51	6.216	
Neutral Acceptance/Neutrality	2018	81	27.02	3.162	0.01^∗^
	2020	85	26.86	4.201	

DAPAS = Death Attitude Profile Assessment Scale.

∗ Student t test for equality of variance, *P* < .05 being considered.

## Discussion

From the beginning of their training process and throughout their professional practice, nurses are constantly in contact with death. They deal with the finitude of life, experience the death of sick people, and end up developing representations about their own death.^3,13^

The fact that nurses’ training is focused on saving lives is an obstacle to the discussion of these issues, and, consequently, to the development of feelings of frustration and suffering, difficulties of acceptance, and consequent distancing of nurses, in the face of a reality increasingly present in the hospital.^3,14^

It should be noted that, in view of the pandemic by COVID-19, this situation was aggravated in the last year.

In view of these experiences, the way of thinking and acting shows aspects such as the nurses’ lack of preparation regarding death and the dying process; the adoption of care models focused on the biomedical, technical, and reductionist paradigm; and the absence of mechanisms to cope with this situation.^15^

In this way, nursing professionals hold a set of knowledge, capacities, and skills that they mobilize in their professional practice for clinical practice contexts, allowing them to consider the health needs of the person or group, and act in all contexts of life, as well as in the face of death and the dying process. Given this unique moment, nursing care for people at the end of life should be characterized by dignity, quality, and satisfaction of needs, trying to avoid feelings of discomfort, anxiety, and/or escape of nurses.^3^ In this sense, regardless of the professional category, it should be noted that nurses’ beliefs are reflected in their attitudes toward death, and may influence practical aspects of their performance.^12^

When analyzing the nurses’ attitudes toward death before and after the first critical period of the pandemic, we found, in 2018, a higher agreement in the dimension Neutral Acceptance/Neutrality, denoting that nurses see death as a part of the life process. Previous studies have already emphasized that nurses should be facilitators throughout this process, effectively responding to the real needs of people at the end of life, involving them in nursing care and their acceptance/rejection.^16,17^

Given that the year 2020 was marked by the SARS-CoV-2 infection, in which the final outcome in several cases led to the death of the person, the nurses in the ES faced this issue even more. Given the pandemic reality, the context of the ES and its mission toward patients, it is possible to realize that nurses continue to view death as a stage of life, because, in their professional practice and care context, they deal with people's clinical instability, requiring quick decision-making, as well as contact with end-of-life situations, resulting from the irreversibility of the person's health condition.^5^

The fact that the mean value obtained in the Neutral Acceptance/Neutrality, after the critical period of the pandemic, is lower, reflects a lower neutral acceptance, compared to the first moment of data collection. The significant number of people with COVID-19 who die in different age groups, not only in old age or with comorbidities, may justify this decrease in the mean value between the 2 periods.^11^

Even though the fear and acceptance as Approach dimensions do not show statistically significant differences, it is important to note the changes in attitude that occurred between the 2 periods.

With regard to fear, it was found that the mean obtained in 2020 was higher than in 2018. This dimension is related to how death has been learned and accepted throughout life,^18^ and can trigger reactions such as uncertainty, suffering, and have an impact on nurses’ professional practice. In a pandemic, which was installed and is still expanding with harmful effects, nurses are faced with patients in a fragile condition and, ultimately, with death. Faced with a virus with high transmissibility, the presence of negative thoughts and anxiety about the possibility of contracting the infection, the occurrence of lesions in physiological processes and the need for complex care,^11,19^ are probable justifications for the increase in the mean between the 2 periods. On the contrary, knowing that the centrality of the nursing profession is caring for others, it is expected that there is a variation in these values, reflecting a feeling of fear with greater expression in the middle of the pandemic, as previously observed.^7^

In Acceptance as Approach, there was a change in attitude between the 2 data collection periods. The mean obtained after the first critical period of the pandemic by COVID-19 was higher compared to 2018. Realizing that the Acceptance as Approach attitude is based on the belief that death constitutes a passage,^12^ the fact that the year 2020 was marked by doubt and uncertainty, nurses made evident, the importance of their religious beliefs and convictions to overcome this pandemic phase and deal with the death of patients. In a previous study, the authors came across the same results, looking at beliefs as a facilitating strategy in coping with death and the dying process.^11^

It is also important to mention that, in both periods, the attitudes acceptance as escape, death becomes an alternative for the end of pain and suffering,^18^ and avoidance, avoiding talking, or thinking about death to reduce anxiety,^18^ did not present significant changes in the 2 moments, relative to the nurses’ answers. Considering the context of the study, where sometimes situations of clinical instability of patients were observed,^5^ the fact that before the pandemic, people died due to clinical worsening may justify the same response pattern of nurses. On the contrary, it should be noted that in the ES, the number of deaths from COVID-19 is not significant, since, if the person's clinical condition is severe, he/she will be admitted to an intensive care unit,^20^ and it is probably in this context that death will occur.

Effectively, dealing with the death of people requires preparation and training, because it is a fragile moment, with feelings of helplessness and frustration.^21^ On the contrary, the pandemic context highlights this issue, as well as nurses’ lack of preparation to face death and the dying process.^7^ Several authors point to the problem of lack of training throughout the academic career and professional life, which can directly influence the nursing care provided.^22,23^

Thus, the need to train nurses in strategies for coping with death and the dying process of sick people is a pressing aspect.^24^ The role of the nurse manager is highlighted as a facilitator in the creation of dialogue strategies and spaces for sharing experiences related to this issue, because these are subjective issues, closely related to the experiences of each nurse.^7^ The institutions themselves should also promote programs that allow their professionals to reflect on these aspects, allowing the negative feelings of this process to be neutralized.^16^ The inclusion of these themes in the initial training of nurses will allow us to break the thinking of a logic focused only on the recovery of life processes and to present death as a possible outcome, throughout the professional performance of each nurse, specifically nurses who work in ER settings.

The limitation of this study is the fact that it only took place in 1 hospital institution and that it is a nonmatched sample. We believe that the initiative developed in this institution, the role of the nurse manager and the involvement of all professionals will contribute positively to rethinking the Approach to death and the dying process.

## Conclusion

The reality of death is an event which is increasingly present in hospital settings. Given their social mandate and the mission of the nursing profession, nurses are the ones who deal directly with death and the dying process of patients; thus, it is important to (re)think about these professionals’ attitudes toward this event. Death, like birth, are stages that integrate the life cycle, but are experienced differently by nurses, and are inevitably reflected in the care provided to the patient.

In this study, there were differences, before and after the first critical period of the pandemic, in the Neutral Acceptance/ Neutrality attitude, with a lower average in the year 2020.

It is known that death is more likely to occur in older people, who are in the final stage of the life cycle. However, in the pandemic context, we observed the death of young people, who were still going through their life cycle, which may justify a lower neutral acceptance of nurses toward death.

There is an urgent need to invest in the training of nurses, namely through training programs developed by the permanent education of the institutions, as well as the creation of spaces for reflection on this issue among the team members, with the collaboration of the nurse manager, aiming at a paradigm shift regarding death and the dying process.

## Conflicts of interest

The authors declare no conflicts of interest.
